# Propagation and reflection of thermoelastic wave in a rotating nonlocal fractional order porous medium under Hall current influence

**DOI:** 10.1038/s41598-023-44712-4

**Published:** 2023-10-17

**Authors:** Farhat Bibi, Hashmat Ali, Ehtsham Azhar, Muhammad Jamal, Iftikhar Ahmed, Adham E. Ragab

**Affiliations:** 1 Department of Mathematics, PMAS Arid Agriculture University, Rawalpindi, Pakistan; 2 Department of Mathematics, COMSATS University Islamabad (CUI), Park Road, Tarlai Kalan, Islamabad 45550 Pakistan; 3 University of Illinois at Urbana-Champaign, Urbana, IL 61801 USA; 4 Industrial Engineering Department, College of Engineering, King Saud University, PO Box 800, Riyadh, 11421 Saudi Arabia

**Keywords:** Mathematics and computing, Physics

## Abstract

This investigation relates to the research on Hall current on propagation and reflection of elastic waves through non-local fractional-order thermoelastic rotating medium with voids. The system is split up into longitudinal and transverse components using the Helmholtz vector rule. It is observed that, through the frequency dispersion relation four coupled quasi-waves exist in the medium. The rotating solid modifies the nature of purely longitudinal and transverse waves toward the quasi-type waves. All the propagating waves are dispersive as they depend upon angular frequency. The quasi-longitudinal wave qP and quasi-transverse wave qSV faces cut-off frequencies. The nonlocal parameter affect all the waves except the quasi void wave. Analytically, the reflection coefficients of the wave are computed using suitable boundary conditions. MATLAB software is used to perform numerical computations for a chosen solid material. The amplitude ratios and the speed of propagation of the wave are plotted graphically for rotational frequency, nonlocal, fractional order, and Hall current parameter. The significant effect of the physical parameters on the computed results has been observed. The cut-off frequency of the waves is also presented graphically. The energy conservation law is proved in the form of energy ratios. The earlier findings in the literature are obtained as special case in the absence of rotation, Hall current parameter and porous voids.

## Introduction

Many fields, including seismology, geophysics, earthquake, engineering etc. have shown interest in the study of reflection and refraction of plane waves. Research on these phenomena is largely extremely important for provision of crucial details about the internal make-up of the earth’s structure and are of huge importance in fields such as acoustic and mining considering the actual uses and theoretical research. Surface reflection and energy partioning have been studied for elastic media^1–4^. Biot’s theory has been the foundation for numerous investigations of transmission of waves in a poroelastic half-space that is saturated.

Yang and Sato^5,6^ used Biot’s theory to check how an earthquake affect a place with saturated soil. Eringen^7^ studied the nonlocal linear elasticity theory and plane waves dispersion. Thermoelasticity theory was first put forth by Biot^8^ which resolves the inconsistency in the uncouple theory, which describe that the temperature is unaffected by elastic charges. The idea of nonlocal theory was given by Eringen^9,10^ and Eringen and Edelen. The core idea of Eringen’s nonlocal theory is that every material in the continuum gets energies that are dispersed due to its extensive correspondence with every other particle in continuum domain. Generalised thermoelastic heat model was added by Sarkar and Tomar^11^ to study the impact of voids and nonlocal parameters on plane wave propagation.

In order to examine a few physical issues involving real materials as polymer, rocks, etc., the concept of fractional calculus is important. Fractional order derivatives can be used to better discuss the chemical characteristics of such a substance. Numerous real-world fields, such as quantum field theory, nuclear physics, control engineering, electromagnetic, chemistry, signal processing, quantum mechanics, astronomy, etc., can benefit from understanding how waves propagate. The basic purpose for using the integer-order models was the lack of a way to solve fractional differential equations. Demirci and Ozlap^12^ discussed an idea about a solution to differential equations involving fractional order. Heat transmission in rotating media with two temperatures and a fractional order Iqbal Kaur et al.^13^ examined the reflection of plane harmonic waves. Sharma and Kumari^14^ investigated how nonlocal fractional-order thermoelastic half space affects plane wave reflection.

Thermoelasticity theories tells about deformation and flow of heat in continuum. Mechanical waves are transmitted when an external force is applied to material body. For example, when a solid is suddenly heated, thermal expansion creates mechanical waves. The study of interactions among the mechanical and thermal fields is the vastest field of continuum dynamics and due to Lorentz forces, Ohm’s principles and other factors, there is a relationship between mechanical and temperature fields. Several actual issues rely on wave propagation in a rotating media. The influence of rotation on plane waves propagating in an elastic solid medium is useful due to its participation in a variety of unique challenges. Most massive entities, including the earth, moon and planets have angular velocities about their polar axes. Also many motion sensors are widely employed in smart weapons systems, cars, robotics and machine control.

Schoenberg and Censors^15^ initiated the investigation for wave propagating in rotating isotropic medium. Recently, Ali et al.^16^ studied the rotational effect in wave reflection by which four quasi waves propagate through the medium. Alesemi^17^ showed how the Coriolis, centrifugal forces and LS forces on the reflection coefficient of plane waves in an anisotropic magneto-thermoelastic rotating with stable angular velocity medium affect the thermal relaxation time. In GN theory of thermoelasticity, Oatman et al.^18^ addressed the deformation of an infinite micro stretch generalized thermoelastic rotating medium under the influence of initially applied magnetic and gravitational field. According to Said^19^, a rotating modified couple stress magneto-thermoelastic medium with two temperatures presents a challenge of fractional derivative heat transfer. Anand^20^ investigated how a spinning orthotropic magneto-thermoelastic solid half space with diffusion reflected plane waves from its free surface. According to the updated Green-Lindsay Model, N. Sarkar and D. Soumen^21^ examined the waves in magneto-thermoelastic materials. Dinesh Kumar et al.^22^ used two relaxation time factors to evaluate the isotropic visco-thermoelastic solid cylinder’s free oscillations in three dimensions. Lata and Kaur^23,24^ also discussed the different theories of thermoelasticity for wave propagation through transversely isotropic magneto-thermoelastic solids.

An induced magnetic field and electric field are created in the elastic medium as a result of the thermal shock and externally applied magnetic field. The resulting electric and magnetic fields produce Hall voltages across the conductors. Since the last ten years, magneto thermoelastic materials have been significant because to the coupling effect between electric and magnetic fields; as a result, the issue of plane wave propagation through these solids is crucial due to their applicability to real-world phenomena. A few physical characteristics of the charge transfer mechanisms in semiconductor materials have recently been studied using the Hall effect. In a semiconductor material, Edwin Hall^25^ found that when a magnetic field perpendicular to the direction of the current was applied, the locations and concentration of electrons deviated from the steady state. A strong magnetic field causes moving charges of electrons and holes, which results in the Hall effect. In their study, Mahdy et al.^26^ examined how the electromagnetic field affected the Hall current as laser pulses left a semiconductor with a fractional thermal order. Lata and Singh^27^ examined how the Hall current was impacted by the electromagnetic field as laser pulses exited a semiconductor with a fractional thermal order.

In the context of Rayleigh waves, Kumar et al.^28^ investigated the connection between Hall current and two temperatures in rotating media. Ehtsham et al.^29^ discussed the effect of Hall current on the reflection phenomenon of magneto-thermoelastic waves in a non-local semiconducting solid. In a transversely isotropic magneto-thermoelastic media, Kaur et al.^30^ studied the effects of Hall current and fractional-order heat transfer caused by ramp heat. In a transversely isotropic magneto thermo elastic media, Parveen Lata and Iqbal Kaur^31^ investigated the effect of Hall current and fractional order heat transfer caused by normal force. The plane wave’s propagation in a rotating visco-thermoelastic medium with Hall current was covered by Kaur et al.^32^. Kumar et al.^33^ considered fractional ordered magneto-micropolar thermo-viscoelastic half-space due to ramp-type heat to check the effects of Hall current and rotation. Porous material or material with voids are terms used to describe material bodies that contain small spaces or pores throughout the body. Because of their beneficial qualities, including their light weight, high specific strength, high surface area, low relative density, thermal and acoustic insulation, and superior permeability, porous materials have a wide range of applications in the fields of aerospace, electronic communications, construction, metallurgy, nuclear energy, petrochemical, mechanical, and environmental protection. Porosity in the medium was initially discussed in the context of traditional work for coupled thermo-elasticity theory by Iesan^34^. By using the Iesan’s model, several researchers have contributed their work in this area. Sing et al.’s^35^ investigation of the effects of porous nonlocal media on wave propagation. Bachher and Sarkar^36^ examined the nonlocal elastic solids with small spaces and fractional derivative heat transport mechanism. Biswas and Sarkar’s description of the waves propagation using the dual-phase-lag model may be found in^37^.

The distribution of pores or voids in porous or void-containing materials is explored by Cowin and Nunziato^38^ and the change in strain and void volume fraction are regarded as independent kinematic variables. On wave propagation in isotropic and anisotropic media with additional factors such as micropolarity, the thermal field with fraction-order derivative, initial stress, voids and diffusion, many scholars have done amazing research work. Ali et al.^39^ investigated the reflection phenomenon of thermo-elastic wave in semiconductor nanostructures non-local porous medium. In order to study the propagation of plane waves, Poonia et al.^40^ employed a nonlocal, transversely isotropic thermoelastic medium with voids and rotation. Ali et al.^41^ examined the reflection phenomenon of thermoelastic wave in a micropolar semiconducting porous medium.

Shishir Gupta et al.^42^ studied the Hall current effect in double poro-thermoelastic material with fractional-order Moore-Gibson-Thompson heat equation subjected to Eringen’s nonlocal theory. Shishir Gupta et al.^43^ discussed photothermal excitation of an initially stressed nonlocal semiconducting double porous thermoelastic material under fractional order triple-phase-lag theory. Shishir Gupta et al.^44^ considered the higher-order fractional and memory response in nonlocal double poro-magneto-thermoelastic medium with temperature-dependent properties excited by laser pulse and Shishir Gupta et al.^45^ studied Double poro-magneto-thermoelastic model with microtemperatures and initial stress under memory-dependent heat transfer. Ramesh Kumar et al.^46^ studied the response of waves in a nonlocal micropolar thermoelastic half-space with voids under dual-phase-lag model, and Devender Sheoran et al.^47^ studied propagation of waves at an interface between a nonlocal micropolar thermoelastic rotating half-space and a nonlocal thermoelastic rotating half-space. Plane waves in an initially stressed rotating magneto-thermoelastic half-space with diffusion and microtemperatures has been discussed by Sheron et al.^48^.

The authors have carefully studied the literature; anyhow, the study on propagation and reflection of elastic waves through non-local fractional-order thermoelastic rotating medium with voids does not exist. Many authors have discussed the same study the under integer order heat conduction model, which is in fact a limiting case in the sense that it is a special case of fractional order analysis. The problem under consideration produces more realistic results as the fractional order study is generalized theory of thermoelasticity. The behavior of temperature can only be well analyzed using the concept of fractional order theory, as temperature strongly dependent upon the fractional time. The Helmholtz vector rule produces frequency dispersion relation, which indicates that four coupled quasi-waves exist in the medium. The rotating solid modifies the nature of purely longitudinal and transverse waves towards quasi-type waves. All the propagating waves are dispersive as they depend upon angular frequency. The quasi-longitudinal wave (*qP*) and quasi-transverse wave (*qSV*) faces cut-off frequencies. The nonlocal parameter affects all the waves except the quasi void wave. Analytically, the reflection coefficients of the wave are computed using suitable boundary conditions. MATLAB software is used to perform numerical computations for a chosen solid material. The amplitude ratios and the speed of propagation of the wave are plotted graphically for rotational frequency, nonlocal, fractional order, and Hall current parameter. The significant effect of the physical parameters on the computed results has been observed. The cut-off frequency of the waves is also presented graphically. The energy conservation law is proved in the form of energy ratios. The earlier findings in the literature are obtained as special case in the absence of rotation, Hall current parameter, and porous voids.

## Basic relation

In this section, some basic constitutive relations are expressed as follows the non-local stress-strain relation is expressed as follows;^49^

1 $$\begin{aligned} \begin{aligned} (1-e^{2}_{1} {\nabla }^{2})\sigma _{ij}=\left[ 2\mu e_{ij} x+\left[ \frac{-\alpha }{K_{T}}T(x)+{\lambda } e_{kk}(x)+{\gamma }{\varphi }\right] {\delta }_{ij}\right] \end{aligned} \end{aligned}$$

where the strain tensor be denoted by

2 $$\begin{aligned} \begin{aligned} e_{ij}=\frac{1}{2}(u_{i,j}+u_{j,i}), \end{aligned} \end{aligned}$$

where $e_{ij}$ is known as strain, $u_{ij}$ is called displacement component. $\nabla ^{2}$ is called the Laplacian operator, ${\alpha }$ is fractional order parameter, $\delta _{ij}$ is Kronecker delta function,$T=T^{\prime }-T_{0}$, T is used to indicate the body’s natural temperature such that $|\frac{T}{T_{0}}|\le 1$ , the Lame’s constants are $\lambda$ and $\mu$, isothermal compressibility is denoted by $K_{T}$ and $T^{\prime }$ represents the material’s amplitude temperature, and $e_{1}={\epsilon }_{0} a_{cl}$ where $\epsilon _{0}$ is a material constant and represents the length of the internal properties. The thermoelastic material’s energy expression for linear theory is denoted by following relation

3 $$\begin{aligned} \begin{aligned} -(1-e^{2}_{1}{\nabla }^{2}){\rho } T_{0}{\eta }=q_{i,i}, \end{aligned} \end{aligned}$$

where $q_{i,i}=-({\rho } C_{e} T+(\frac{\alpha T_{0}}{K_{T}})e_{ij})$ is the expression for motion without body forces and $C_{e}$ represents the specific heat. When there are no body forces, the equation of motion for a nonlocal isotropic thermoelastic solid with porous voids can be expressed as

4 $$\begin{aligned} \begin{aligned} {\sigma }_{ij,j}={\rho }{\ddot{u}}_{i}, \end{aligned} \end{aligned}$$

In the thermoelastic material’s nonlocal heat transfer is

5 $$\begin{aligned} \begin{aligned} (1-e^{2}_{1}{\nabla }^{2})\left( 1+{\tau _{0}}\frac{{\partial }^{\alpha }}{\partial t^{\alpha }}\right) q_{i}=K{\nabla }T, \end{aligned} \end{aligned}$$

where $\tau _{0}$ is the relaxation time and K is the thermal conductivity, $\rho$ is the density. The fractional order parameter is defined as follows^14^;

6 $$\begin{aligned}{} & {} \frac{{\partial }^{\alpha }}{{\partial }t^{\alpha }}f(x,t)= {\left\{ \begin{array}{ll} f(x,t)-f(x,0) &{} \alpha \rightarrow 0\\ l^\frac{(1-\alpha ) \partial f(x,t)}{\partial t} &{} 0<\alpha <1\\ \frac{\partial f(x,t)}{\partial t} &{} \alpha \rightarrow 1. \end{array}\right. } \end{aligned}$$

7 $$\begin{aligned}{} & {} \begin{aligned} l^{\alpha }f(x,t)=\frac{1}{\Gamma ({\alpha })}{\int _{0}^{1}(t-g)^{\alpha -1}f(x,g)dg}, \end{aligned} \end{aligned}$$

where $\Gamma$ is constant and represents the gamma function so that 0 ${\le }{\alpha }{\le } 1$. Using the universal Ohm’s law and taking into account the impact of Hall current on a medium with fixed conductivity $\sigma _{0}$^24^ the current density is expressed as follows;

8 $$\begin{aligned} \begin{aligned} \bar{J}=\frac{\sigma _{0}}{1+m^{2}}\left[ \bar{E}+{\mu }_{0}\left( \frac{\partial \bar{u}}{\partial t} \times \bar{H}-\frac{\bar{J}}{e n_{e}} \times \bar{H}_{0}\right) \right] , \end{aligned} \end{aligned}$$

Equation of motion in rotating frame of reference is obtained by putting Eqs. (1–3) into Eqs. (4) and (5)

9 $$\begin{aligned}{} & {} \begin{aligned} {\mu }{\nabla }^{2}\varvec{u}+({\mu }+{\lambda })\nabla ({\nabla }.\varvec{u})-\frac{\alpha }{K_{T}}{\nabla }T+\gamma {\nabla }{\varphi }={\rho }(1-e^{2}_{1}{\nabla }^{2})\left[ \ddot{\varvec{u}}+\varvec{\Omega }\times (\varvec{\Omega }\times {\varvec{u}})+2{\varvec{\Omega }}\times \dot{\varvec{u}}\right] . \end{aligned} \end{aligned}$$

10 $$\begin{aligned}{} & {} \begin{aligned} \left( 1+{\tau }_{0}\frac{{\partial }^{\alpha }}{{\partial t}^{\alpha }}\right) \left( C_{e} \dot{T}+\frac{{\alpha }T_{0}}{\rho K_{T}}{\nabla }.\dot{u}+mT_{0}\dot{\varphi }\right) =\frac{K}{\rho }{\nabla }^{2}T. \end{aligned} \end{aligned}$$

Similarly, equation of voids is taken from^39^

11 $$\begin{aligned} \begin{aligned} {\alpha }^{\prime }{\nabla }^{2}{\varphi }-{\upsilon }{\varphi }-{\tau }^{\prime } \dot{\varphi }-{\gamma }{\nabla }.{u}+mT={\rho }{\chi }(1-e^{2}_{1}{\nabla }^{2}){\ddot{\varphi }} \end{aligned} \end{aligned}$$

### Special case

When ${\alpha }\rightarrow {0}$, relation (5) reducing to the classical theory of coupled thermoelasticity and when integer order ${\alpha }\rightarrow {1}$, the Lord and Shulman thermoelasticity hypothesis can be obtained from Eq. (5).

## Mathematical modeling of the problem

We consider the semiconducting nanostructure medium with finite conductivity and fractional-order three phase lag model (TPL) thermoelastic model with constant temperature $T_{0}$ as initial thermo-elasticity, a magnetic field that is initially applied, so that $\bar{H}_{0}=(0,\bar{H}_{0},0)$, $\bar{u}_{0}=(\bar{u}_{1},0,\bar{u}_{2})$ along the y direction. Furthermore, the plane of propagation for the waves is chozen as the xz-plane. The displacement vector $\bar{u}$ has components , it is also supposed that $\bar{E}=0$. The current densities $J_{1}$ and $J_{3}$ using Eq. (8) are computed as:

12 $$\begin{aligned}{} & {} \begin{aligned} J_{1}=\frac{{\sigma }_{0}{\mu _{0}}{H}_{0}}{1+m^{2}}\left( {m}\frac{\partial u}{\partial t}-\frac{\partial w}{\partial t}\right) \end{aligned} \end{aligned}$$

13 $$\begin{aligned}{} & {} \begin{aligned} J_{3}=\frac{{\sigma }_{0}{\mu _{0}}{H}_{0}}{1+m^{2}}\left( \frac{\partial u}{\partial t}+{m}\frac{\partial w}{\partial t}\right) \end{aligned} \end{aligned}$$

Equation (9–10) are transformed to following component form.

14 $$\begin{aligned}{} & {} \begin{aligned} & ({\lambda }+2{\mu })\frac{{\partial }^{2}u}{{\partial }x^{2}}+({\lambda }+{\mu })\frac{{\partial }^{2}w}{{\partial }x{\partial }z}+{\mu }\frac{{\partial }^{2}u}{{\partial }z^{2}}-{\beta }_{T}\frac{\partial T}{\partial x}-(1-e^{2}_{1}{\nabla }^{2}){\mu _{0}}{J_{3}}{H_{0}}+{\gamma }\frac{{\partial }{\varphi }}{{\partial }x}= {\rho }(1-e^{2}_{1}{\nabla }^{2})\\&\left( \frac{{\partial }^{2}u}{{\partial }t^{2}}-{\Omega }^{2}u+2{\Omega }\frac{{\partial }w}{{\partial }t}\right) , \end{aligned} \end{aligned}$$

15 $$\begin{aligned}{} & {} \begin{aligned} & ({\lambda }+2{\mu })\frac{{\partial }^{2}w}{{\partial }z^{2}}+({\lambda }+{\mu })\frac{{\partial }^{2}u}{{\partial }x{\partial }z}+{\mu }\frac{{\partial }^{2}w}{{\partial }x^{2}}-{\beta }_{T}\frac{\partial T}{\partial z}+(1-e^{2}_{1}{\nabla }^{2}){\mu _{0}}{J_{1}}{H_{0}}+{\gamma }\frac{{\partial }{\varphi }}{{\partial }z}= {\rho }(1-e^{2}_{1}{\nabla }^{2})\\&\left( \frac{{\partial }^{2}w}{{\partial }t^{2}}-{\Omega }^{2}w-2{\Omega }\frac{{\partial }u}{{\partial }t}\right) , \end{aligned} \end{aligned}$$

16 $$\begin{aligned}{} & {} \begin{aligned} \left( 1+{\tau }_{0}\frac{{\partial }^{\alpha }}{{\partial t}^{\alpha }}\right) \left( C_{e} \dot{T}+\frac{{\alpha }T_{0}}{\rho K_{T}}{\nabla }.\dot{u}+mT_{0}\dot{\varphi }\right) =\frac{K}{\rho }{\nabla }^{2}T \end{aligned} \end{aligned}$$

17 $$\begin{aligned}{} & {} \begin{aligned} {\alpha }^{\prime }{\nabla }^{2}{\varphi }-{\upsilon }{\varphi }-{\tau }^{\prime } \dot{\varphi }-{\gamma }{\nabla }.{u}+mT={\rho }{\chi }(1-e^{2}_{1}{\nabla }^{2}){\ddot{\varphi }}, \end{aligned} \end{aligned}$$

Since Eqs. (14) and (15) are coupled equations therefore, we apply following decomposition rule of Helmholtz is employed to uncouple the system

18 $$\begin{aligned} \begin{aligned} \varvec{u}={\nabla }{\phi }+{\nabla } \times \varvec{\psi },\quad {\nabla }.\varvec{\psi }=\varvec{0}. \end{aligned} \end{aligned}$$

where scalar potential ${\phi }(x,z,t)$ yields an irrotational vector field and vector potential $\varvec{\psi }(x,z,t)$ produces a solenoid vector field. The model can be made non-dimensional with the aid of the following set of quantities:

19 $$\begin{aligned} \begin{aligned} & C_{T}{w}^{*}({\bar{x}},{\bar{\phi }},{\bar{z}},{\bar{w}},{\bar{\psi }})=({x},{\phi },{z},{w},{\psi }),\ {w}^{*}({\bar{t}},{\bar{\tau _{0}}})=(t,{\tau _{0}}), \ \frac{T}{\bar{T}_{0}}={\bar{T}}, \ {\nabla }^{2}=\frac{\bar{\nabla }^{2}}{(w^{*}{C_{T}})^{2}}, \\&{\varphi }=\frac{(w^{*}{C_{T}})^{2}}{\chi }{\bar{\varphi }}, \ {\bar{\alpha }^{\prime }}=\frac{{\alpha }^{\prime }}{{\rho }{\chi }{C_{T}}^{2}}, \ {\gamma }=\frac{\bar{\gamma }}{w{*}},\ {\Omega }=\frac{\bar{\Omega }}{w^{*}}, \ M=\frac{\mu ^{2}_{0} H^{2}_{0} \sigma _{0} w^{*}}{\rho },\ C^{2}_{T}=\frac{\lambda +2\mu }{\rho },\\&\beta _{1}=\frac{\beta _{T} T_{0} w^{*}}{\rho C_{T}},\ \beta _{2}=\frac{\alpha }{K_{T}}, \ \alpha _{1}=\frac{(w^{*})^{2} C_{T}}{\rho \chi }, \ \beta ^{2}=\frac{\rho C^{2}_{T}}{\mu },\ \alpha _{2}=\frac{\beta _{2}}{\rho w^{*} C_{T} C_{e}},\ w^{*}=\frac{k}{\rho C_{e} C^{2}_{T}},\\&\beta _{3}=\frac{m (w^{*})^{2} C^{2}_{T}}{\chi C_{e}}. \end{aligned} \end{aligned}$$

Making use of relation (18), the governing dimensionless system (14–17) in terms of potential functions becomes (for simplicity omitting the bar sign) as follows:

20 $$\begin{aligned}{} & {} \begin{aligned} & \left[ {\nabla }^{2}-(1-e^{2}_{1}{\nabla }^{2})\left( \frac{{\partial }^{2}}{{\partial }t^{2}}+\left( \frac{M}{1+m^{2}}\right) \frac{\partial }{{\partial }t}-{\Omega }^{2}\right) \right] {\phi }+{\alpha }_{1}{\varphi }+(1-e^{2}_{1}{\nabla }^{2})\\&\left[ \left( \frac{Mm}{1+m^{2}}\right) \frac{\partial }{{\partial }t}+2{\Omega }\frac{\partial }{{\partial }t}\right] {\psi } -{\beta }_{1}T=0, \end{aligned} \end{aligned}$$

21 $$\begin{aligned}{} & {} \begin{aligned} & \left[ {\nabla }^{2}-{\beta }^{2}(1-e^{2}_{1}{\nabla }^{2})\left( \frac{{\partial }^{2}}{{\partial }t^{2}}+\left( \frac{M}{1+m^{2}}\right) \frac{\partial }{{\partial }t}-{\Omega }^{2}\right) \right] {\psi }-{\beta }^{2}(1-e^{2}_{1}{\nabla }^{2})\\&\left[ \left( \frac{Mm}{1+m^{2}}\right) \frac{\partial }{{\partial }t}+2{\Omega }\right] {\phi }=0, \end{aligned} \end{aligned}$$

22 $$\begin{aligned}{} & {} \begin{aligned} \left( 1+{\tau }_{0}(w^{*})^{(1-\alpha )}\frac{{\partial }^{\alpha }}{{\partial t}^{\alpha }}\right) \left[ \frac{\partial T}{\partial t}+{\alpha _2}{\nabla }^{2}\frac{\partial \phi }{\partial t}+{\beta _3}\frac{\partial \varphi }{\partial t}\right] ={\nabla }^{2}T. \end{aligned} \end{aligned}$$

23 $$\begin{aligned}{} & {} \begin{aligned} \alpha ^{\prime }{\nabla }^{2}{\varphi }-\frac{\upsilon ({w^{*}})^{2}}{\rho \chi } \varphi -\frac{\tau ^{\prime }w^{*}}{\rho \chi } \frac{\partial \varphi }{\partial t}-\frac{\gamma }{\rho {w^{*}}C_{T}^{2}}\nabla ^{2}\phi +\frac{m T_{0}T}{\rho C_{T}^{2}}=(1-e_{1}^{2}\nabla ^{2})\frac{\partial ^{2} \varphi }{\partial t^{2}} \end{aligned} \end{aligned}$$

It can be observed that set of Eqs. (20–23) are coupled in four functions $\phi , \psi , T, \varphi$. In the next section, the solution of the coupled system will be calculated.

## Propagation of waves

The following harmonic wave solution can be used to satisfy the wave equations:

24 $$\begin{aligned} \begin{aligned} (\phi ,\psi , T, \varphi )=(\bar{\phi },\bar{\psi },\bar{T},\bar{\varphi })exp(i \xi (x sin{\theta }+zcos{\theta })-i \omega t) \end{aligned} \end{aligned}$$

Where $\bar{\phi },\bar{\psi },\bar{T}$ and $\bar{\varphi }$ are constant amplitudes of propagating waves, $\omega$ is the angular frequency, $\theta$ is angle of propagation vector, $\xi$ represents the wave number and the vector constant, where $r = (xi + yj + zk)$ is the position vector. By substituting relation (24), Eqs. (20–23) are transformed to following algebraic system of linear equation.

25 $$\begin{aligned}{} & {} \begin{aligned} ({\xi }^{2}-(1+e_{1}^{2}{\xi }^{2})C_{1})\bar{\phi }+(1+e_{1}^{2}{\xi }^{2})C_{2}{\bar{\psi }}+\beta _{1} {\bar{T}}-\alpha _{1}{\bar{\varphi }}=0, \end{aligned} \end{aligned}$$

26 $$\begin{aligned}{} & {} \begin{aligned} ({\xi }^{2}-(1+e_{1}^{2}{\xi }^{2})C_{4})\bar{\psi }+(1+e_{1}^{2}{\xi }^{2})C_{3}{\bar{\phi }}=0, \end{aligned} \end{aligned}$$

27 $$\begin{aligned}{} & {} \begin{aligned} (-C_{6}+{\xi }^{2})\bar{T}+C_{5}{\xi }^{2}\bar{\phi }-C_{7}{\bar{\varphi }}=0, \end{aligned} \end{aligned}$$

28 $$\begin{aligned}{} & {} \begin{aligned} \left( -C_{10}{\xi }^{2}-C_{11}+i\omega {C_{12}}+(1+e_{1}^{2}{\xi }^{2}){\omega }^{2}\right) {\bar{\varphi }}+{C_{8}}{\xi }^{2}{\bar{\phi }}+C_{9}{\bar{T}}=0, \end{aligned} \end{aligned}$$

Where

29 $$\begin{aligned} \begin{aligned} & C_{1}=\left( {\omega }^{2}+i{\omega }\left( \frac{M}{1+m^{2}}\right) +\Omega ^{2}\right) , \ C_{2}=\left( i {\omega }\left( \frac{Mm}{1+m^{2}}\right) +{2 i \Omega \omega }\right) ,\\&\ C_{3}={\beta }^{2}\left( {2 \Omega }-\frac{Mmi{\omega }}{1+m^{2}}\right) , \ C_{4}={\beta }^{2} \left( {\omega }^{2}+i {\omega }(\frac{Mm}{1+m^{2}})+\Omega ^{2}\right) ,\\&\ C_{5}=\left( 1+{\tau _{0}}({w}^{*})^{(1-\alpha )}({-i \omega ^{(\alpha )}}) \right) ({i \omega \alpha _{2}}), \ C_{6}=\left( 1+{\tau _{0}}({w}^{*})^{(1-\alpha )}({-i \omega ^{(\alpha )}}) \right) ({i \omega }),\\&\ C_{7}=\left( 1+{\tau _{0}}({w}^{*})^{(1-\alpha )}({-i \omega ^{(\alpha )}}) \right) ({i \omega \beta _{3}}), \ C_{8}=\frac{\gamma }{\rho w^{*} C_{T}^{3}}, \ C_{9}=\frac{m T_{0}}{\rho C_{T}^{2}}, \ C_{10}=\alpha ^{\prime },\\&\ C_{11}=\frac{\upsilon (w^{*})^{2}}{\rho \chi }, \ C_{12}=\frac{{\tau }^{\prime }w^{*}i \omega }{\rho \chi }. \end{aligned} \end{aligned}$$

For unknown $\bar{\phi }, \bar{\psi }, \bar{T}$ and $\bar{\varphi }$ the non-trivial solution to homogenous linear system (25–28) exist if the coefficient matrix’s determinant vanishes,

30 $$\begin{aligned} \begin{aligned} Q_{1}({\xi }^{8})+Q_{2}({\xi }^{6})+Q_{3}({\xi }^{4})+Q_{4}({\xi }^{2})+Q_{5}=0, \end{aligned} \end{aligned}$$

where

$$Q_{1}=-C_{10} C_{1}C_{10} e_{1}^{2}+C_{10}C_{4}e_{1}^{2}+C_{10}C_{2}C_{3}e_{1}^{4}-C_{1}C_{10}C_{4}e_{1}^{4}+ e_{1}^{2} \omega ^{2}-C_{1}e_{1}^{4} \omega ^{2}-C_{4}e_{1}^{4} \omega ^{2}-C_{2}C_{3} e_{1}^{6} \omega ^{2}+C_{1}C_{4}e_{1}^{6} \omega ^{2},$$

$$\begin{aligned}Q_{2} & = C_{1}C_{10}-C_{11}+C_{10}C_{4}+\beta _{1}C_{10}C_{5}+C_{10}C_{6}+ \alpha _{1}C_{8}+C_{1}C_{11}e_{1}^{2}+2 C_{10}C_{2} C_{3} e_{1}^{2}\\&\quad -2 C_{1} C_{10} C_{4} e_{1}^{2}+C_{11}C_{4} e_{1}^{2}-\beta _{1} C_{10} C_{4} C_{5} e_{1}^{2}-C_{1}C_{10}C_{6} e_{1}^{2}-C_{10} C_{4} C_{6} e_{1}^{2}-\alpha _{1} C_{4} C_{8}e_{1}^{2} \\&\quad + C_{11}C_{2} C_{3} e_{1}^{4}-C_{1} C_{11} C_{4} e_{1}^{4}-C_{10} C_{2}C_{3} C_{6} e_{1}^{4}+C_{1} C_{10} C_{4} C_{6} e_{1}^{4}+C_{12} i \omega -C_{1} C_{12} e_{1}^{2} i \omega \\&\quad -C_{12} C_{4} e_{1}^{2} i\omega -C_{12} C_{2} C_{3}e_{1}^{4} i\omega +C_{1} C_{12} C_{4} e_{1}^{4}i \omega +\omega ^{2}-2 C_{1} e_{1}^{2} \omega ^{2}-2 C_{4} e_{1}^{2}\omega ^{2}-\beta _{1} C_{5} e_{1}^{2} \omega ^{2} \\&\quad -C_{6} e_{1}^{2} \omega ^{2}-3 C_{2} C_{3} e_{1}^{4} \omega ^{2}+3 C_{1} C_{4}e_{1}^{4}\omega ^{2}+\beta _{1} C_{4} C_{5} e_{1}^{4} \omega ^{2} +C_{1} C_{6} e_{1}^{4} \omega ^{2}+C_{4} C_{6} e_{1}^{4} \omega ^{2}\\&\quad +C_{2}C_{3} C_{6} e_{1}^{6} \omega ^{2}-C_{1} C_{4} C_{6}e_{1}^{6} \omega ^{2},\end{aligned}$$

$$\begin{aligned}Q_{3} & = C_{1} C_{11}+C_{10}C_{2} C_{3}-C_{1} C_{10} C_{4}+C_{11} C_{4}+\beta _{1} C_{11}C_{5}-\beta _{1}C_{10}C_{4} C_{5}-C_{1} C_{10} C_{6}\\ & \quad +C_{11}C_{6}-C_{10} C_{4} C_{6} -\alpha _{1}C_{4} C_{8}-\alpha _{1} C_{6}C_{8}+\beta _{1} C_{7}C_{8}-\alpha _{1}C_{5} C_{9}-C_{7} C_{9}\\ & \quad +2C_{11}C_{2} C_{3}e_{1}^{2}-2 C_{1} C_{11}C_{4} e_{1}^{2}-\beta _{1} C_{11} C_{4}C_{5}e_{1}^{2}-C_{1}C_{11} C_{6}e_{1}^{2}-2 C_{10}C_{2}C_{3} C_{6}e_{1}^{2}\\ & \quad+2C_{1} C_{10}C_{4} C_{6}e_{1}^{2}-C_{11} C_{4} C_{6} e_{1}^{2}+\alpha _{1} C_{4} C_{6} C_{8} e_{1}^{2}-\beta _{1} C_{4} C_{7} C_{8} e_{1}^{2}+\alpha _{1} C_{4} C_{5}C_{9}e_{1}^{2}\\ & \quad+C1 C_{7}C_{9}e_{1}^{2}+C_{4} C_{7}C_{9}e_{1}^{2} - C_{11} C_{2} C_{3} C_{6}e_{1}^{4}+C_{1} C_{11}C_{4}C_{6} e_{1}^{4}+ C_{2}C_{3}C_{7}C_{9} e_{1}^{4}\\ & \quad-C_{1} C_{4}C_{7}C_{9} e_{1}^{4}-C_{1}C_{12}i\omega -C_{12} C_{4}i\omega -\beta _{1} C_{12}C_{5}i\omega -C_{12} C_{6}i\omega -2C_{12} C_{2} C_{3} e_{1}^{2}i\omega \\ & \quad +2 C_{1}C_{12} C_{4}e_{1}^{2}i\omega +\beta _{1} C_{12} C_{4} C_{5} e_{1}^{2}i\omega +C_{1}C_{12}C_{6}e_{1}^{2}i\omega +C_{12} C_{4}C_{6} e_{1}^{2}i\omega +C_{12} C_{2}C_{3}C_{6} e_{1}^{4}i\omega \\ & \quad -C_{1}C_{12}C_{4} C_{6} e_{1}^{4}i\omega -C_{1}\omega ^{2}-C_{4} \omega ^{2}-\beta _{1} C_{5} \omega ^{2}-C_{6}\omega ^{2}-3 C_{2} C_{3} e_{1}^{2}\omega ^{2}+3 C_{1} C_{4}e_{1}^{2} \omega ^{2}\\ & \quad +2\beta _{1} C_{4} C_{5} e_{1}^{2} \omega ^{2}+2 C_{1} C_{6} e_{1}^{2} \omega ^{2}+2C_{4} C_{6} e_{1}^{2} \omega ^{2}+3 C_{2} C_{3} C_{6} e_{1}^{4}\omega ^{2}-3 C_{1} C_{4}C_{6}e_{1}^{4}\omega ^{2},\end{aligned}$$

$$\begin{aligned}Q_{4} & = C_{11}C_{2}C_{3}-C_{1}C_{11}C_{4}-\beta _{1} C_{11}C_{4} C_{5}-C_{1} C_{11}C_{6}- C_{10} C_{2}C_{3}C_{6}+ C_{1}C_{10} C_{4}C_{6}\\ &\quad -C_{11}C_{4}C_{6}+ \alpha _{1}C_{4}C_{6}C_{8} -\beta _{1}C_{4} C_{7}C_{8}+ \alpha _{1}C_{4}C_{5}C_{9}+C_{1}C_{7}C_{9}+ C_{4}C_{7}C_{9} \\ &\quad -2C_{11}C_{2}C_{3}C_{6}e_{1}^{2} + 2 C_{1} C_{11}C_{4} C_{6} e_{1}^{2} +2 C_{2}C_{3}C_{7} C_{9} e_{1}^{2} -2 C_{1}C_{4} C_{7}C_{9}e_{1}^{2} - C_{12}C_{2}C_{3}i\omega \\ &\quad+ C_{1}C_{12} C_{4} i\omega + \beta _{1} C_{12} C_{4}C_{5} i\omega + C_{1}C_{12}C_{6}i\omega +C_{12}C_{4} C_{6}i\omega + 2 C_{12} C_{2} C_{3}C_{6}e_{1}^{2}i\omega \\ &\quad - 2 C_{1}C_{12} C_{4}C_{6}e_{1}^{2} i\omega -C_{2}C_{3}\omega ^{2}+C_{1}C_{4}\omega ^{2}+ \beta _{1}C_{4} C_{5} \omega ^{2}+ C_{1}C_{6}\omega ^{2} +C_{4}C_{6}\omega ^{2} \\ &\quad + 3 C_{2}C_{3}C_{6}e_{1}^{2} \omega ^{2}-3 C_{1} C_{4}C_{6}e_{1}^{2} \omega ^{2},\end{aligned}$$

$$Q_{5}=-C_{11}C_{2} C_{3} C_{6}+C_{1} C_{11}C_{4} C_{6}+ C_{2}C_{3}C_{7}C_{9}- C_{1} C_{4}C_{7} C_{9}+ C_{12} C_{2}C_{3}C_{6}i\omega -C_{1}C_{12}C_{4}C_{6} i\omega +C_{2} C_{3} C_{6} \omega ^{2}-C_{1}C_{4} C_{6}\omega ^{2}.$$

For the propagation of plane waves in nonlocal thermoelastic solid media, Equation (30) provides the dispersion relation, which corresponds to speed of propagations. The relation (30) gives eight roots of $\xi$ such that $\pm \xi _{1},\ \pm \xi _{2},\ \pm \xi _{3},\ \pm \xi _{4}$. In the medium, there are four waves that corresponds to four positive values of $+\xi$, $i=1,2,3,4$. Let these waves are quasi longitudinal wave (*qP*-wave), thermal wave (*qT*-wave), quasi transverse wave (*qSV*-wave) and void wave (*qV*-wave). The expression $Q_{1},Q_{2},Q_{3},Q_{4},Q_{5}$ are complex valued, therefore, speed of the wave is a complex quantity such that $\xi =\xi _{r}+i \xi _{i}$, where $\xi =\xi _{r}$ is real part and $\xi =\xi _{i}$ is imaginary part. Moreover, its real part ($\xi =\xi _{r}$) gives speed of propagation and the imaginary part ($\xi =\xi _{i}$) gives attenuation coefficient. The rotation in the solid disturbs the isotropy and makes it like anisotropic. The anisotropic behavior of the solid converts the purely longitudinal and transverse type of the waves into quasi-longitudinal and quasi-transverse waves.

### Special case of the model

In the absence of the rotation, Hall current and voids i.e. $\Omega = m= \alpha ^{\prime }= \upsilon =\tau ^{\prime }= \gamma =\chi =0$ then Eqs. (9) and (10) are transformed as follows:

31 $$\begin{aligned}{} & {} \begin{aligned} ((v^{2}+e_{1}^{2}\omega ^{2})-V_{T}^{2}+A_{1})\bar{\phi }-\beta \bar{T}=0, \end{aligned} \end{aligned}$$

32 $$\begin{aligned}{} & {} \begin{aligned} \omega ^{2}\tau _{0}^{*}\beta T_{0} \bar{\phi }-\left( \frac{K}{\rho }- C_{e} v^{2}\tau _{0}^{*} \right) \bar{T}=0, \end{aligned} \end{aligned}$$

The non-trivial solution of equations yields the same quadratic polynomial as obtained by Subani and Aangeeta^14^. Furthermore, it can be seen that Eq. (21) becomes un-coupled in potential $\bar{\psi }$ corresponding to *SV*-wave. Its speed of propagation is computed as follows;

33 $$\begin{aligned} \begin{aligned} v_{SV}=\sqrt{V_{s}-e_{1}^{2}\omega ^{2}}, \end{aligned} \end{aligned}$$

Equation (33) is same as Eq. (32) obtained by Subani and Aangeeta^14^. The SV wave’s speed is only dependent upon the non-local parameter and angular frequency. The speed becomes non-dispersive in the local medium as it does not depend upon the wave frequency.

## Reflection of qP wave

**Figure 1:**
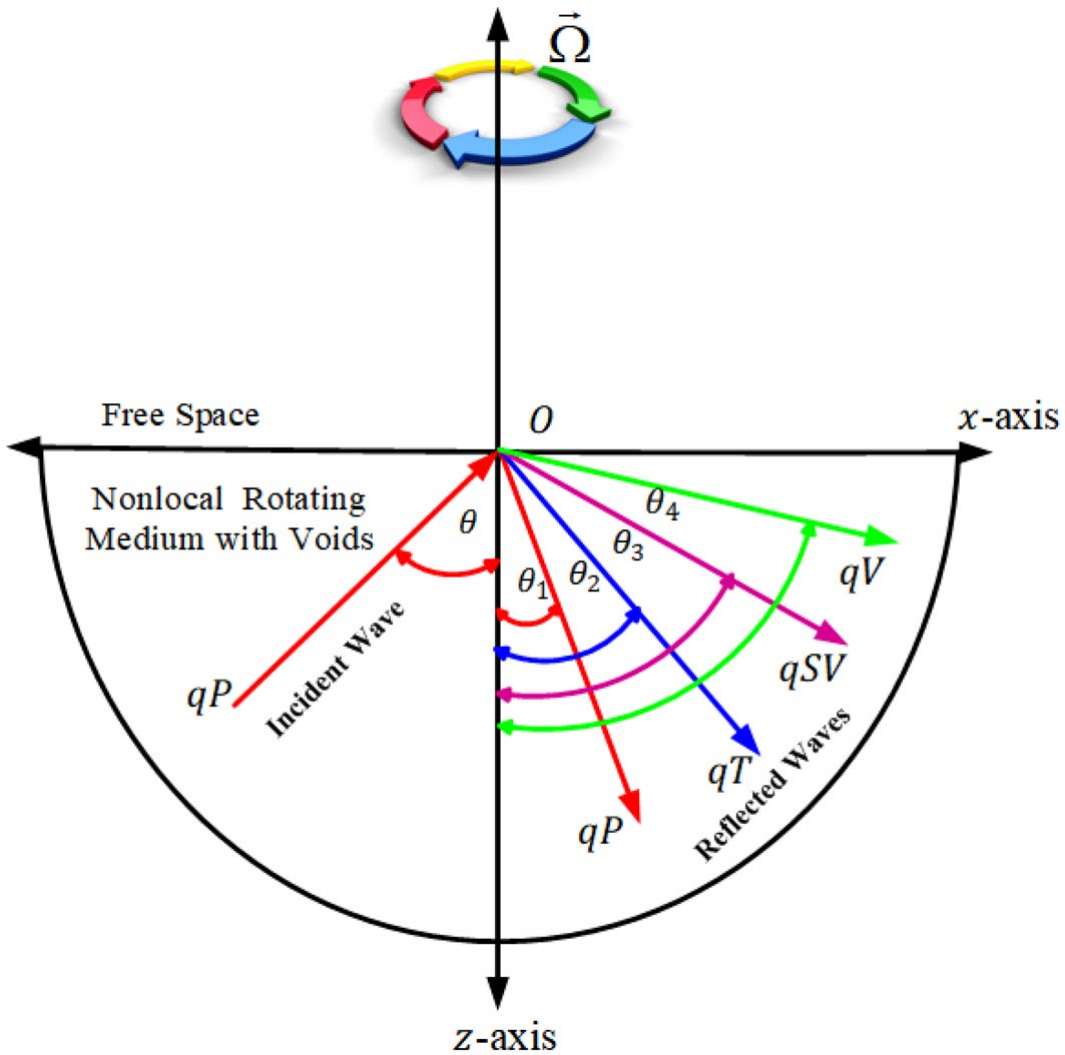
Reflection view of the waves at the free boundary of the solid.

Let quasi-longitudinal wave (*qP*-wave) be the incident wave at the free boundary resulting in generation of four reflected waves named as *qP*, *qT*, *qSV*, and *qV*-wave as shown in Fig. (1). The incident wave makes an angle of incidence $(\theta )$ with respect to the normal. The appropriate potentials of reflected and incident waves are taken into consideration as

34 $$\begin{gathered} \phi = A_{0} e^{{i\xi _{0} (xsin\theta + zcos\theta ) - i\omega t}} + A_{1} e^{{i\xi _{1} (xsin\theta _{1} - zcos\theta _{1} ) - i\omega t}} + A_{2} e^{{i\xi _{2} (xsin\theta _{2} - zcos\theta _{2} ) - i\omega t}} + \hfill \\ \;A_{3} e^{{i\xi _{3} (xsin\theta _{3} - zcos\theta _{3} ) - i\omega t}} + A_{4} e^{{i\xi _{4} (xsin\theta _{4} - zcos\theta _{4} ) - i\omega t}} \hfill \\ \end{gathered}$$

35 $$\begin{gathered} T = \zeta _{0} A_{0} e^{{i\xi _{0} (xsin\theta + zcos\theta ) - i\omega t}} + \zeta _{1} A_{1} e^{{i\xi _{1} (xsin\theta _{1} - zcos\theta _{1} ) - i\omega t}} + \zeta _{2} A_{2} e^{{i\xi _{2} (xsin\theta _{2} - zcos\theta _{2} ) - i\omega t}} + \hfill \\ \;\zeta _{3} A_{3} e^{{i\xi _{3} (xsin\theta _{3} - zcos\theta _{3} ) - i\omega t}} + \zeta _{4} A_{4} e^{{i\xi _{4} (xsin\theta _{4} - zcos\theta _{4} ) - i\omega t}} \hfill \\ \end{gathered}$$

36 $$\begin{aligned}{} & {} \begin{aligned} & {\psi }=\eta _{0}A_{0} e^{i \xi _{0}(xsin{\theta }+zcos{\theta })-i \omega t}+\eta _{1}A_{1} e^{i \xi _{1}(xsin{\theta _{1}}-zcos{\theta _{1}})-i \omega t}+\eta _{2}A_{2} e^{i \xi _{2}(xsin{\theta _{2}}-zcos{\theta _{2}})-i \omega t}+\\&\eta _{3}A_{3} e^{i \xi _{3}(xsin{\theta _{3}}-zcos{\theta _{3}})-i \omega t}+\eta _{4}A_{4} e^{i \xi _{4}(xsin{\theta _{4}}-zcos{\theta _{4}})-i \omega t} \end{aligned} \end{aligned}$$

37 $$\begin{gathered} \varphi = l_{0} A_{0} e^{{i\xi _{0} (xsin\theta + zcos\theta ) - i\omega t}} + l_{1} A_{1} e^{{i\xi _{1} (xsin\theta _{1} - zcos\theta _{1} ) - i\omega t}} + l_{2} A_{2} e^{{i\xi _{2} (xsin\theta _{2} - zcos\theta _{2} ) - i\omega t}} + \hfill \\ \;l_{3} A_{3} e^{{i\xi _{3} (xsin\theta _{3} - zcos\theta _{3} ) - i\omega t}} + l_{4} A_{4} e^{{i\xi _{4} (xsin\theta _{4} - zcos\theta _{4} ) - i\omega t}} \hfill \\ \end{gathered}$$

Where coupling parameters are defined as follows;

38 $$\begin{gathered} \eta _{i} = - \frac{{\left( {1 + e_{1}^{2} \xi _{i}^{2} } \right)C_{3} }}{{\xi _{i}^{2} - \left( {1 + e_{1}^{2} \xi _{i}^{2} } \right)C_{4} }},~\zeta _{i} = \frac{{\left( { - C_{6} + \xi _{i}^{2} } \right)\left( { - \xi _{i}^{2} + \left( {1 + e_{1}^{2} \xi _{i}^{2} } \right)C_{1} - \left( {1 + e_{1}^{2} \xi _{i}^{2} } \right)C_{2} \eta _{i} } \right) + C_{5} \xi _{i}^{2} }}{{\left( {C_{6} - \xi _{i}^{2} } \right)\frac{{\alpha _{1} }}{{\beta _{1} }} + C_{7} }}, \hfill \\ \;l_{i} = \frac{{ - \xi _{i}^{2} + \left( {\left( {1 + e_{1}^{2} \xi _{i}^{2} } \right)C_{1} - \left( {1 + e_{1}^{2} \xi _{i}^{2} } \right)C_{2} \eta _{i} } \right) + \alpha _{1} \zeta _{i} }}{{\beta _{1} }},~\;i = 0,1,2,3,4. \hfill \\ \end{gathered}$$

Now we employ the following boundary conditions which are necessary to solve the suggested problem. Because there is no stress at the boundary surface at $z=0$ and thermally insulated, we get,

39 $$\begin{aligned} \begin{aligned} \sigma _{zx}=0,\ \sigma _{zz}=0,\ \frac{\partial T}{\partial z}=0,\\ \text {change in fractional volume of the void is zero, i.e.}\\ \frac{\partial \varphi }{\partial z}=0 \quad at \quad z=0. \end{aligned} \end{aligned}$$

The boundary conditions (39) are transformed to the following system of algebraic equation with the help of Eqs. (34–37), the following system of algebraic equation is obtained

40 $$\begin{aligned}{} & {} \begin{aligned} \left( \frac{A_{1}}{A_{0}}\right) c_{11}+\left( \frac{A_{2}}{A_{0}}\right) c_{12}+\left( \frac{A_{3}}{A_{0}}\right) c_{13}+\left( \frac{A_{4}}{A_{0}}\right) c_{14}=d_{1}, \end{aligned} \end{aligned}$$

41 $$\begin{aligned}{} & {} \begin{aligned} \left( \frac{A_{1}}{A_{0}}\right) c_{21}+\left( \frac{A_{2}}{A_{0}}\right) c_{22}+\left( \frac{A_{3}}{A_{0}}\right) c_{23}+\left( \frac{A_{4}}{A_{0}}\right) c_{24}=-d_{2}, \end{aligned} \end{aligned}$$

42 $$\begin{aligned}{} & {} \begin{aligned} \left( \frac{A_{1}}{A_{0}}\right) c_{31}+\left( \frac{A_{2}}{A_{0}}\right) c_{32}+\left( \frac{A_{3}}{A_{0}}\right) c_{33}+\left( \frac{A_{4}}{A_{0}}\right) c_{34}=d_{3}, \end{aligned} \end{aligned}$$

43 $$\begin{aligned}{} & {} \begin{aligned} \left( \frac{A_{1}}{A_{0}}\right) c_{41}+\left( \frac{A_{2}}{A_{0}}\right) c_{42}+\left( \frac{A_{3}}{A_{0}}\right) c_{43}+\left( \frac{A_{4}}{A_{0}}\right) c_{44}=d_{4}, \end{aligned} \end{aligned}$$

Where

44 $$\begin{aligned} \begin{aligned} & c_{1i}=(sin2{\theta _{i}}-\eta _{i}cos2{\theta _{i}})\xi ^{2}_{i}, ~ c_{2i}=(\lambda +2\mu cos^{2}\theta _{i}+\eta _{i} \mu sin2{\theta _{i}})\xi ^{2}_{i}+\beta \rho \zeta _{i}-\gamma l_{i},\\&c_{3i}=\xi _{i} \zeta _{i} cos{\theta _{i}}, ~ c_{4i}=\xi _{i} l_{i} cos{\theta _{i}}, ~ d_{1}=(sin2{\theta }-\eta _{0}cos2{\theta })\xi ^{2}_{0}, \\&d_{2}=-(\lambda +2\mu cos^{2}\theta +\eta _{0} \mu sin2{\theta })\xi ^{2}_{0}+\beta \rho \zeta _{0}-\gamma l_{0}, ~ d_{3}=\xi _{0} \zeta _{0} cos{\theta }, ~ d_{4}=\xi _{0} l_{0} cos{\theta }. \end{aligned} \end{aligned}$$

## Energy conservation

In this section, we discuss the energy conservation of the system. The computed results can be validated in the context of energy conservation. Following^50^, the energy transmission rate per unit area is given by

45 $$\begin{aligned} \begin{aligned} \qquad \qquad \qquad \qquad \qquad \qquad \qquad \tilde{P}=\sigma _{zz} \dot{w}+\sigma _{zx} \dot{u} \end{aligned} \end{aligned}$$

Defining the quantities $E_{1}, E_{2}, E_{3}$ and $E_{4}$ as energy ratio corresponding to reflected *qP*, *qT*, *qSV* and *qV* wave to the incident wave. These energy ratios are computed as follows:

46 $$\begin{aligned} \begin{aligned} \qquad \qquad \qquad \qquad \qquad \qquad E_{i}=\frac{<P_{i}>}{<P_{0}>},\quad i=1,2,3,4. \end{aligned} \end{aligned}$$

Where

$$P_{i}=(c_{1i}) \omega \sin \theta _{i} |\frac{A_{i}}{A_{0}}|^{2}+(c_{2i}) \omega \cos \theta _{i}|\frac{A_{i}}{A_{0}}|^{2}$$,   $$\frac{A_{i}}{A_{0}}=G_{i}$$

and using the values of $c_{1i}$ and $c_{2i}$ the expression of energy becomes

47 $$\begin{aligned} \begin{aligned} & P_{i}=(\sin 2\theta _{i}-\eta _{i}\cos 2\theta _{i})\xi _{i}^{2} \omega \sin \theta _{i}|G_{i}|^{2}+[(\lambda +2\mu \cos ^{2}\theta _{i}+\eta _{i}\mu \sin 2\theta _{i})\xi _{i}^{2}+\beta \rho \zeta _{i}- \gamma l_{i} ] \omega \cos \theta _{i}|G_{i}|^{2}\\&P_{0}=(\sin 2\theta -\eta _{0}\cos 2\theta )\xi _{0}^{2} \omega \sin \theta |G_{0}|^{2}+[(\lambda +2\mu \cos ^{2}\theta +\eta _{0}\mu \sin 2\theta )\xi _{0}^{2} +\beta \rho \zeta _{0}-\gamma l_{0} ] \omega \cos \theta |G_{0}|^{2} \end{aligned} \end{aligned}$$

## Graphical discussion of the analytical results

Now the computed results are studied graphically under certain physical parameters. The following values of copper like material’s elastic constants are taken from^14^ for this purpose.

$\lambda =3.64 \times 10^{10} N/m^{2},\ \mu = 5.46 \times 10^{10} N/m^{2}, \ \tau _{0}=0.007682,~ \beta _{T}=293,\ w^{*}=8655,\ \mu _{0}=0.7, \ H_{0}=0.06, \ \sigma _{0}=0.3,\ \rho =2.33 \times 10^{10} kg/m^{3},\ \beta =0.97,\ KT=386,\ C_{e}=695 J/(kg.K), \ T_{0}=800 K.$ and voids parameters are given by^51^ $\upsilon =1.2 \times 10^{10} Pa.m^{2},\ \alpha ^{\prime }=8 \times 10^{10} Pa.m^{2}, \ \tau ^{\prime }=10^{6} Pa.s,\ \gamma =1 Pa,\ \chi =0.16 kg/m^{3}.$

In the following paragraphs, the impact of different physical parameters including non-local parameter, rotational frequency, fractional order, and Hall current parameter on the speed of wave propagation and their corresponding amplitude ratios is studied.

### Effect of nonlocal parameter on propagation speed

**Figure 2:**
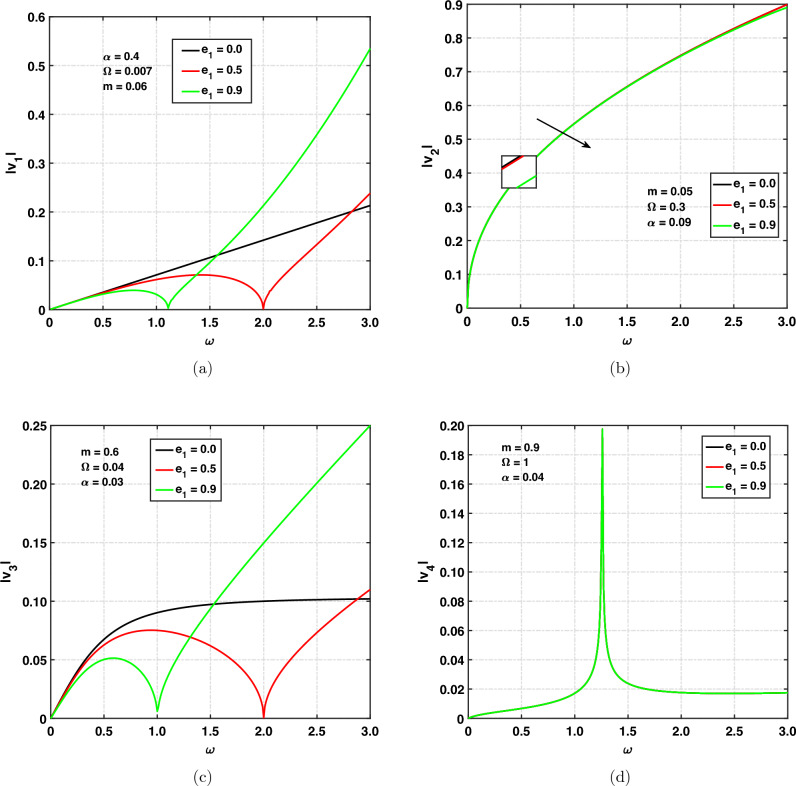
Speed of propagation versus $\omega$ for nonlocal parameter.

Figure 2a,d show the variation of speed of the quasi longitudinal wave (*qP*), quasi thermal wave (*qT*), quasi transverse wave (*qSV*) and quasi void wave (*qV*) versus angular frequency $\omega$ for different values of the nonlocal parameter. The chosen nonlocal parameters are 0, 0.5, and 0.9. Keeping in view its physical significance that it is in fact a characteristic length of the solid, the value of $e_{1}=0$ corresponds to local medium (classical medium), and $e_{1}\ne 0$ corresponds to the nonlocal nature of the solid. Figure 2a shows the effect of $e_{1}$ on $|v_{1}|$ by taking $\alpha =0.4, m=0.06$. And furthermore, a solid is rotating with $\Omega =0.007$. Here, we discuss the speed of the *qP* wave. For $e_{1}=0.0$, the curve is increasing slowly with increasing values of angular frequency, for $e_{1}\rightarrow 0.5$ curve is firstly increasing slowly and then decreasing sy with respect to $\omega$ (when $0<\omega <2.0$ ). When $\omega \rightarrow 2.0$, $|v_{1}|$ is zero. For $\omega >2.0$ curve shows increasing behavior. And shows the cut-off frequency at $\omega =2.0$. For the nonlocal parameter $e_{1}=0.9$, the curve is increasing and then decreasing, and it has been observed that when $\omega \rightarrow 1.1$, $|v_{1}|$ is zero. Afterwards, for $\omega >1.1$ the curve is exponentially increasing. And shows the cut-off frequency at $\omega =1.1$. So the speed of the *qP* wave is decreasing with increasing values of the nonlocal parameter. Figure 2b graphically displays the speed of quasi thermal wave (*qT*) propagation. It can be seen that speed is exponentially increasing with increasing values of angular frequency. The speed of the quasi thermal wave (*qT*) is decreasing with increasing values of non-local parameter. The speed is maximum for lower values of angular frequency, and it has maximum values at $\omega =3$. Figure 2c shows the effect of $e_{1}$ on $|v_{3}|$ by taking $\alpha =0.03, m=0.6$. And furthermore, a solid is rotating with $\Omega =0.04$. For $e_{1}=0.0$, the curve is increasing with increasing values of angular frequency and then shows a constant line with respect to $\omega$ ($\omega >0.8$). It means that when $\omega >0.8$, the speed of the *qSV* wave is independent of angular frequency. For $e_{1}=0.5$, the curve is firstly increasing and then decreasing with respect to $\omega$ (when $0<\omega <2.0$). When $\omega \rightarrow 2.0$, $|v_{3}|$ is zero. For $\omega >2.0$, the curve shows increasing behavior. And shows cut-off frequency when $\omega \rightarrow 2.0$. For the nonlocal parameter $e_{1}=0.9$, the curve is increasing, and it has been observed that when $\omega \rightarrow 1.0$, $|v_{3}|$ is zero. Afterwards, for $\omega >1.0$, the curve is exponentially increasing. And shows the cut-off frequency when $\omega \rightarrow 2.0$. So the speed of the *qSV* wave is decreasing with increasing values of the nonlocal parameter.

The effect of $e_{1}$ on $|v_{4}|$ is studied by taking $\alpha =0.04, m=0.9$ in Fig. 2d. And furthermore, a solid is rotating with $\Omega =1$. The speed of the quasi void wave (*qV*-wave) under different values of the nonlocal parameter is increasing when $0<\omega <1.4$ and decreasing when $\omega >1.4$. A spike has been observed at $\omega =1.4$. Physically, spikes mean that the magnitude is reaching infinity. The amplitude of a wave is too high means approaching to infinity. The speed of the quasi void wave is independent of the non-local parameter.

### Effect of rotational frequency on propagation speed

**Figure 3:**
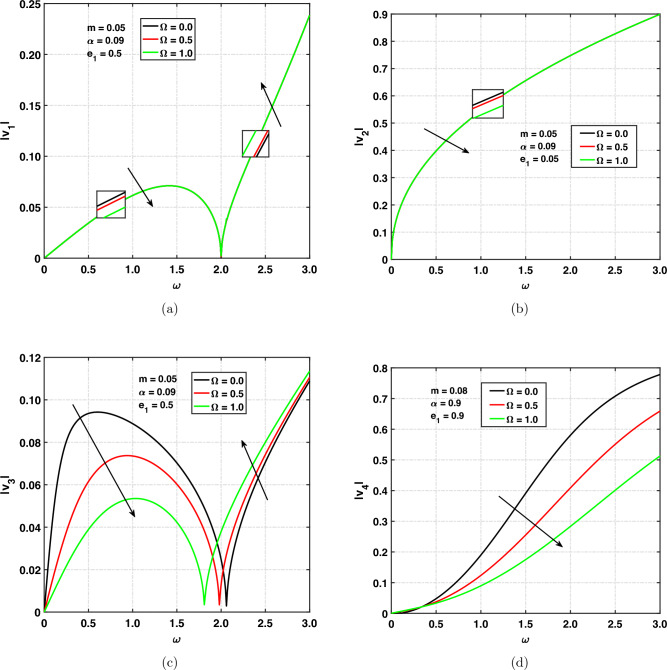
Speed of propagation versus $\omega$ for rotational frequency.

Figure 3a,d, show the variation of speed for rotating and non-rotating medium versus angular frequency. Where $\Omega =0$ corresponds to non-rotating medium and $\Omega \ne 0$ corresponds to rotating medium. The chosen values of $\Omega$ are 0, 0.5, 1.0. Figure 3a illustrates the quasi-longitudinal wave’s (*qP*) speed propagation and indicates the effect of $\Omega$ on $|v_{1}|$ is studied by taking the $\alpha =0.05, m=0.05, e_{1}=0.5$. The speed of the *qP* wave is decreasing or moving down with increasing values of rotational frequency when $0<\omega <2.0$. For $\omega >2.0$, speed is increasing with increasing values of rotational frequency. Curves are first increasing with respect to angular frequency and then decreasing. It has been observed that when $\omega \rightarrow 2.0$, $|v_{1}|$ is zero. After that, for $\omega >2.0$, curves are increasing exponentially. It means that the cut-off frequency in the range of 0 to 2.0 due to rotation speed decreases, and reverse effect detected beyond the cut-off frequency. Figure 3b graphically represents the speed of quasi thermal wave (*qT*) propagation. It can be seen that the speed is exponentially increasing with increasing values of angular frequency. The speed of the quasi thermal wave (*qT*) is decreasing with increasing values of rotational frequency. The speed is maximum for lower values of rotational frequency.

Figure 3c illustrates the (*qSV*) wave speed propagation and shows the effect of $\Omega$ on $|v_{3}|$ by taking the $\alpha =0.09, m=0.05, e_{1}=0.5$. The speed of the *qSV* wave is decreasing under different values of $\Omega$ when the angular frequency is between 0 and almost 2.0, and then increasing under different values of $\Omega$ when the angular frequency exceeds 2.0. It has been observed that at $\omega =2.0$, $|v_{3}|$ is closed to 0. It means that the cut-off frequency in the range of 0 to 2.0 due to rotation speed decreases, but beyond the cut-off frequency, rotation increases the speed of propagation. Graphical representation of the quasi void wave (*qV*-wave) propagation is shown in Fig. 3d. It can be seen that speed is increasing with respect to angular frequency in rotating and non-rotating medium. The speed of the quasi void wave (*V*-wave) is decreasing with increasing values of rotational frequency. The speed is maximum for lower values of rotational frequency. Since the speed of each wave depends on the angular frequency, we concluded that all waves are dispersive.

### Effect of fractional order on propagation speed

**Figure 4:**
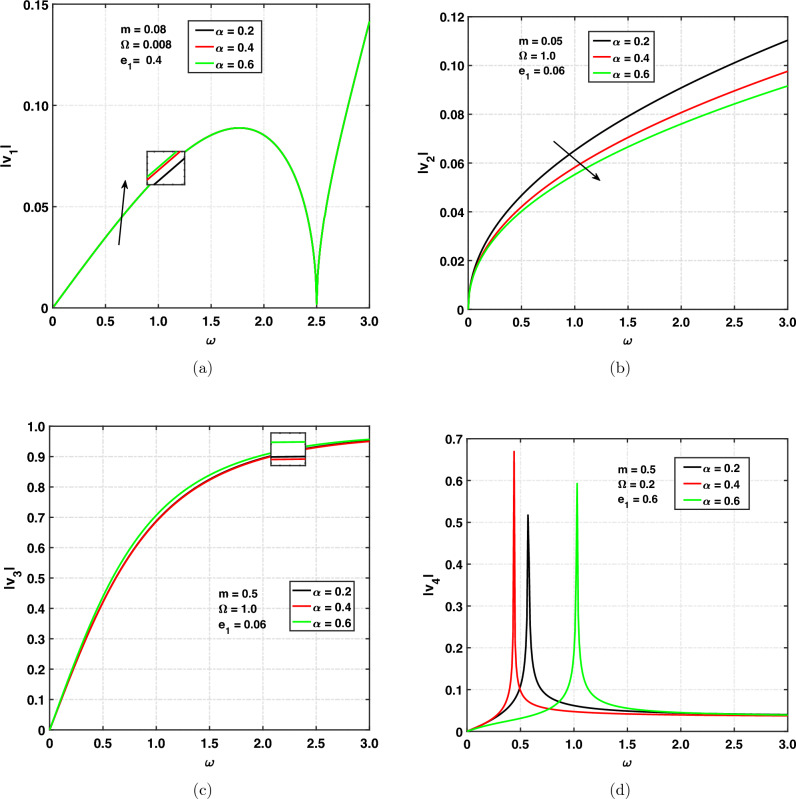
Speed of propagation versus $\omega$ for fractional order parameter.

In Fig. 4a,d, the propagation speed of the *qP*, *qT*, *qSV*, and *qV* waves for different values of fractional order versus angular frequency is shown. The fractional order parameter’s value must be between 0 and 1. When we take $\alpha =1$, fractional order study becomes integer order study. The values we choose here are 0.2, 0.4, and 0.6. Figure 4a illustrates the quasi-longitudinal wave’s (*qP*) speed propagation and shows the effect of $\alpha$ on $|v_1|$ by taking the $m=0.08, e_1=0.4, \Omega =0.008$. The speed of the *qP* wave is increasing or moving up with increasing values of fractional order. Curves are first increasing with respect to angular frequency and then decreasing. It has been observed that at $\omega =2.5$, $|v_1|$ is zero. After that, for $\omega >2.5$, curves are increasing exponentially, and the speed of the *qP* wave is increasing with respect to angular frequency. It means that the cut-off frequency in the range of 0 to 2.5 due to rotation speed increases, and same effect is beyond the cut-off frequency. Figure 4b graphically displays the speed of quasi thermal wave (*qT*) propagation. It can be seen that the speed is increasing with respect to the angular frequency. The speed of the *qT* wave is decreasing with increasing values of fractional order. The speed is maximum for lower values of fractional order. The speed of the *qSV* wave propagation is displayed in Fig. 4c. It can be seen that the speed of the *qSV* wave is increasing with respect to the angular frequency. Since the speed is frequency dependent. So it is called a dispersive wave. The speed of the *qSV* wave is decreasing when fractional order increasing from 0.2 to 0.4 and increasing when fractional order exceeds 0.4.

Figure 4d represents the quasi void wave’s (*qV*-wave) speed propagation, and the effect of $\alpha$ on $|v_4|$ is studied by taking the $m=0.5, \Omega =0.2, e_{1}=0.6$. It can be seen that for some frequencies, it shows increasing and decreasing behavior and spikes have been observed at some particular frequencies. Physically, spikes mean that magnitude is showing to infinity. The highest amplitude has been observed at $\alpha =0.4$. For $\alpha =0.2$, a spike has been observed at $\omega =0.6$. For $\alpha =0.4$, a spike has been observed at $\omega =0.4$. For $\alpha =0.6$, a spike has been observed at $\omega =1.1$. This shows that spikes shift to the origin when fractional order increases from 0.2 to 0.4 and move away from the origin when fractional order exceeds 0.4.

### Effect of Hall current on propagation speed

**Figure 5:**
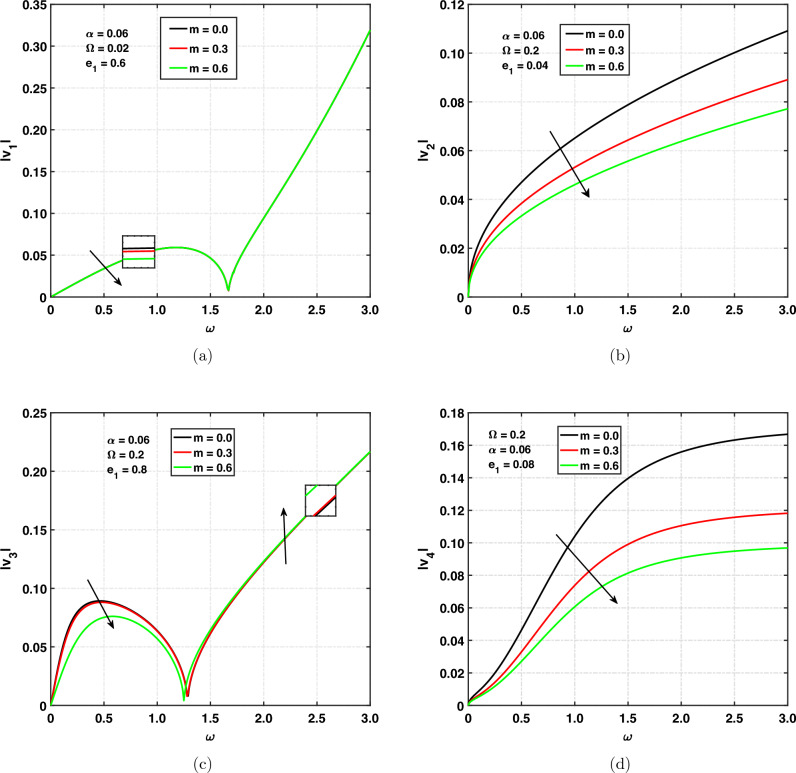
Speed of propagation versus $\omega$ for Hall current.

Figure 5a,d illustrate the propagation speed of the *qP*, *qT*, *qSV* and *qV* wave for different values of Hall current parameter versus angular frequency. The values that are selected for Hall current are $m=0.0,0.3,0.6$. Figure 5a illustrates the quasi longitudinal wave’s (*qP*) speed propagation and shows the effect of $``m''$ on $|v_{1}|$ by taking the $\alpha =0.06, \Omega =0.02, e_{1}=0.6$. The speed of the *qP* wave is decreasing with increasing values of the Hall current parameter. And observed cut-off frequency at $\omega =1.6$. Curves are first increasing with respect to the angular frequency and then decreasing. It has been observed that when $\omega \rightarrow 1.6$, $|v_{1}|$ is zero. After that, for $\omega >1.6$, curves are increasing exponentially. It means that the cut-off frequency in the range of 0 to 1.6 Hall current parameter decreases the speed of the *qP* wave and has same effect beyond this range. The speed of quasi thermal wave (*qT*) propagation is shown in Fig. 5b. It can be seen that the speed is increasing with respect to the angular frequency. The speed of the *qT* wave is decreasing with increasing values of the Hall current parameter. The speed reaches its maximum at $m=0.0$. Figure 5c indicates the quasi-transverse wave’s (*qSV*) speed propagation and shows the effect of $``m''$ on $|v_{3}|$ by taking the $\alpha =0.06, \Omega =0.2, e_{1}=0.04$. The speed of the *qSV* wave is decreasing or moving down with increasing values of the Hall current parameter $(0<\omega <1.3)$. It shows the cut-off frequency at $\omega =1.3$. For $\omega >1.3$, speed is increasing with increasing values of the Hall current parameter. Curves are first increasing with respect to the angular frequency and then decreasing. After that, for $\omega >1.3$, curves are increasing exponentially. It means that the cut-off frequency in the range of 0 to 1.3 due to the Hall current parameter speed decreases, and the opposite effect is beyond the cut-off frequency. The speed of propagation of the quasi void wave (*qV*-wave) is represented in Fig. 5d, which shows that the speed is increasing with respect to the angular frequency. So it is called the dispersive wave. The speed of the *qV* wave is decreasing with increasing values of the Hall current parameter.

### Effect of nonlocal parameter on amplitude ratios

**Figure 6:**
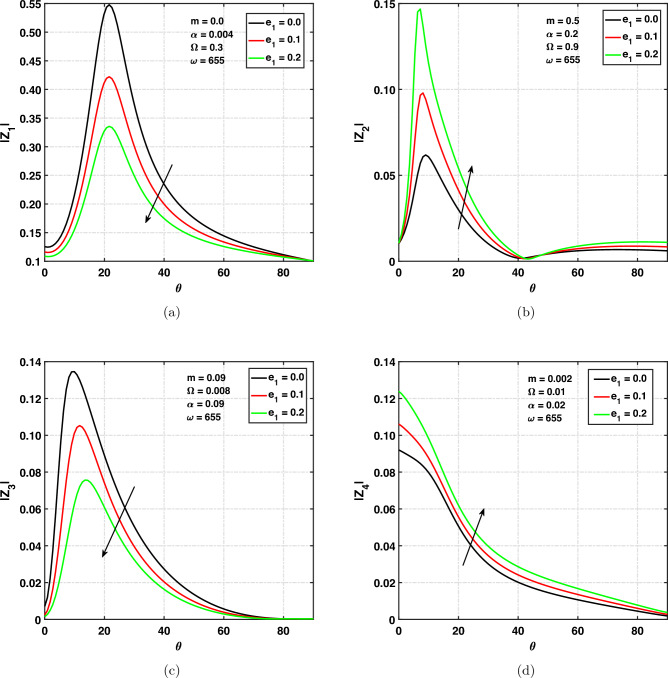
Amplitude ratios versus angle of incidence $\theta$ under non-local parameter.

Figure 6a,d show the variation of amplitude ratios of the (*qP*), (*qT*), (*qSV*), and *qV* waves versus angle of incidence $0^{\circ } \le \theta \le 90^{\circ }$ for different values of nonlocal parameter. The non-local parameter’s values are chosen to be 0.0, 0.1, and 0.2 which compares the local and non-local parameters as well. The $e_{1}=0$ corresponds to local theory (classical theory), and $e_{1}\ne 0$ corresponds to nonlocal theory. In Fig. 6a the impact of non-local parameter on amplitude ratios is shown. To check the effect, some values of the parameter have been fixed. The amplitude ratio of the *qP* wave is decreasing with increasing nonlocal parameter’s values. It means that the amplitude ratio is dependent on a nonlocal parameter. Amplitude ratio is increasing with respect to the $\theta$ $(0^{\circ }\le \theta \le 25^{\circ })$ and decreasing with respect to the $\theta$ when $(\theta \ge 25^{\circ })$ till grazing incidence. The amplitude ratio has a peak value at $\theta =25^{\circ }$. Finally, at $\theta =\frac{\pi }{2}$, the amplitude ratios decrease to zero.

Figure 6b represents the variation of $\alpha$ on $|z_{2}|$ by taking fixed values of $\Omega =0.9, m=0.5, e_{1}=0.08, \omega =655$. It indicates that with increasing non-local parameter’s values, the amplitude ratio is increasing. It means that the amplitude ratio of the *qT* wave is dependent on local and non-local theories. We also noticed that for $\theta =45^{\circ }$, $|z_{2}|=0$. Therefore, $\theta =45^{\circ }$ acts as a critical angle for the existence of the *qT* wave. The same effect of non-local theory on $|z_{2}|$ is for $\theta \ge 45^{\circ }$ till $\theta =\frac{\pi }{2}$. Figure 6c demonstrates the effect of the non-local parameter on $|z_{3}|$. To check the effect, some values of the parameter have been fixed. The amplitude ratio of the *qSV* wave is decreasing with increasing nonlocal parameter’s values. It means that the amplitude ratio is dependent on nonlocal parameter. The amplitude ratio is increasing with respect to the $\theta$ $(0^{\circ }\le \theta \le 10^{\circ })$ and decreasing with respect to $\theta$ when $(\theta \ge 10^{\circ })$ till grazing incidence. The amplitude ratio has its peak value at $\theta =10^{\circ }$. One can observe from Fig. 6d that with increasing values of the non-local parameter, the amplitude ratio of quasi void wave is increasing. The amplitude ratio of the *qV* Wave is decreasing with respect to the angle of incidence ($\theta$). Finally, at grazing incidence angles, the amplitude ratio decreases to zero.

### Effect of rotational frequency on amplitude ratio

**Figure 7:**
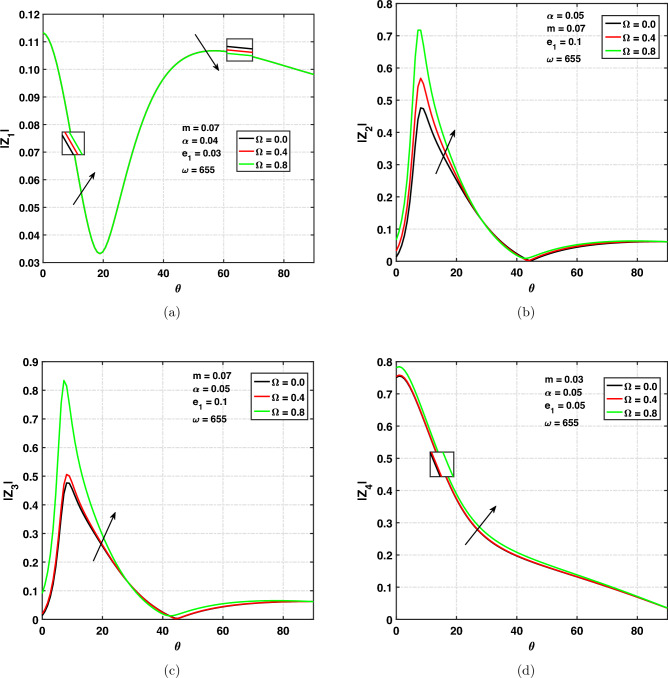
Amplitude ratios versus angle of incidence $\theta$ in rotating and non-rotating medium.

Figure 7a,d show the variation of amplitude ratios of the (*qP*), (*qT*), (*qSV*) and *qV* waves versus angle of incidence $0^{\circ } \le \theta \le 90^{\circ }$ under different values of rotational frequency. The values of rotational frequency are chosen to be 0.0, 0.4, and 0.8. This in fact, gives a comparison between rotating and non-rotating medium. The $\Omega =0$ corresponds to a non-rotating medium, and $\Omega \ne 0$ corresponds to rotating medium. One can see from Fig. 7a the effect of $\Omega$ on $|z_{1}|$ by taking fixed values of parameters. The amplitude ratio of the *qP* wave is increasing under increasing values of rotational frequency $(0^{\circ }\le \theta \le 20^{\circ })$ and decreasing when $\theta \ge 20^{\circ }$. Curves are decreasing with respect to the angle of incidence $(0^{\circ }\le \theta \le 20^{\circ })$ and increasing with respect to $\theta$ $(20^{\circ }\le \theta \le 60^{\circ })$ and again slightly decreasing when $\theta \ge 60^{\circ }$ till grazing incidence. Figure 7b indicates the variation of $\Omega$ on $|z_{2}|$ by taking fixed values of $\alpha =0.05, m=0.07, e_{1}=0.1, \omega =655$. It indicates that with increasing values of rotational frequency, the amplitude ratio is increasing. It means that the amplitude ratio of the *qT* is dependent on rotating and non-rotating mediums. We also noticed that for $\theta =\frac{\pi }{4}$, $|z_{2}|=0$. Therefore, $\theta =45^{\circ }$ acts as a critical angle for the existence of the *qT* wave. The same effect of the rotational frequency on $|z_{2}|$ is for $\theta \ge 45^{\circ }$ till $\theta =\frac{\pi }{2}$.

The effect of $\Omega$ on $|z_{3}|$ is studied by taking fixed values of $\alpha =0.05, m=0.07, e_{1}=0.1, \omega =655$ in Fig. 7c. It indicates that with increasing values of rotational frequency, the amplitude ratio is increasing. It means that the amplitude ratio is dependent on the rotating and non-rotating mediums. We also noticed that for $\theta =45^{\circ }$, $|z_{3}|=0$. Therefore, $\theta =45^{\circ }$ act as a critical angle for the existence of the *qSV* wave. The same effect of the rotational frequency on $|z_{3}|$ is for $\theta \ge 45^{\circ }$ till grazing angle of incidence. One can observe from Fig. 7d the impact of $\Omega$ on the amplitude ratios of the quasi void wave. The amplitude ratio of the *qV* wave is increasing when the rotational frequency is increasing. We can see that the amplitude ratio is decreasing with increasing values of $\theta$.

### Effect of fractional order on amplitude ratios

**Figure 8:**
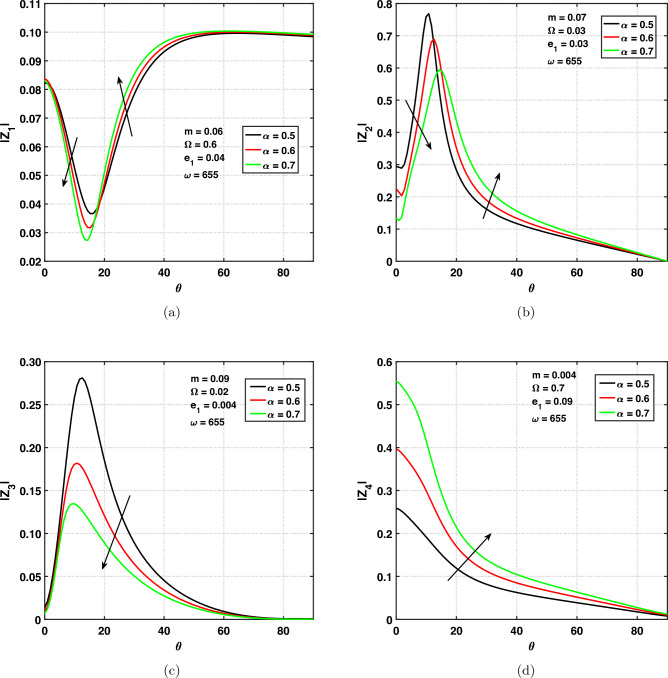
Amplitude ratios versus angle of incidence $\theta$ for fractional order.

In Fig. 8a,d, the variation of amplitude ratios of the (*qP*), (*qT*), (*qSV*) and *qV* waves verses angle of incidence $0^{\circ } \le \theta \le 90^{\circ }$ under different values of fractional order parameter is shown. Different values of fractional order are selected to be 0.5, 0.6, and 0.7. Keeping in view that for $\alpha =1$, the current study may produce the result of integer order wave theory. For fractional order investigation, $\alpha$ should be chosen as $0<\alpha <1$. One can see from Fig. 8a the effect of $\alpha$ on $|z_{1}|$ by taking fixed values of various parameters. The amplitude ratio of the *qP* wave is decreasing under increasing values of fractional order $(0^{\circ }\le \theta \le 15^{\circ })$ and increasing when $\theta \ge 15^{\circ }$. Curves are decreasing with respect to the angle of incidence $(0^{\circ }\le \theta \le 15^{\circ })$ and increasing with respect to $\theta$ $(15^{\circ }\le \theta \le 60^{\circ })$ and show a constant line when $\theta \ge 60^{\circ }$ till grazing incidence.

The amplitude ratio of the *qT* wave is graphically represented in Fig. 8b, which shows that $z_{2}$ decreases for increasing values of fractional order $(0^{\circ } \le \theta \le 10^{\circ })$ afterwards, increasing with increasing values of $\alpha$ $(\theta \ge 10^{\circ })$ till grazing angle of incidence. Amplitude ratio is increasing with respect to $\theta$ $(0^{\circ }\le \theta \le 10^{\circ })$ and decreasing with respect to $\theta$ when $(\theta \ge 10^{\circ })$ till $\theta =\frac{\pi }{2}$. The amplitude ratio has its peak value at $\theta =10^{\circ }$. The effect of the fractional order on amplitude ratios is shown in Fig. 8c. To check the effect, some parameter’s values have been fixed. The amplitude ratio of the *qSV* wave is decreasing with increasing values of the fractional order parameter. It means that the amplitude ratio is dependent on the fractional order parameter. Amplitude ratio is increasing with respect to $\theta$ $(0^{\circ }\le \theta \le 10^{\circ })$ and decreasing with respect to $\theta$ when $(\theta \ge 10^{\circ })$ till $\theta = \frac{\pi }{2}$. The amplitude ratio has its peak value at $\theta =10^{\circ }$. One can observe from Fig. 8d the impact of $\alpha$ on the amplitude ratio of the quasi void wave. The amplitude ratio of the *qV* wave is increasing when the fractional order is increasing. We can see that amplitude ratio is decreasing with increasing values of $\theta$. Finally, at grazing incidence angles, the amplitude ratio decreases to zero.

### Effect of Hall current on amplitude ratios

**Figure 9:**
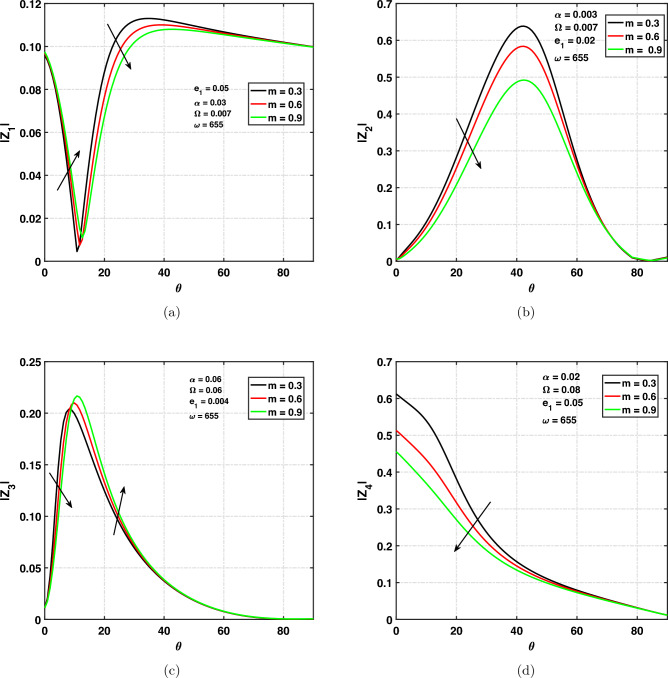
Amplitude ratios versus angle of incidence $\theta$ under Hall current parameter.

Figure 9a,d show the effect of amplitude ratios of (*qP*), (*qT*), (*qSV*) and *qV*-waves verses angle of incidence $0^{\circ } \le \theta \le 90^{\circ }$ under different values of parameter (Hall current). Different values of Hall current are chosen to be $m=0.3,0.6,0.9$. One can see from Fig. 9a the effect of $``m''$ on $|z_{1}|$ by taking the fixed values of various parameters. The amplitude ratio of the *qP* wave is increasing under increasing values of the Hall current parameter $(0^{\circ }\le \theta \le 10^{\circ })$, and reverse effect detected when $\theta \ge 10^{\circ }$. Curves are decreasing with respect to the angle of incidence $(0^{\circ }\le \theta \le 10^{\circ })$ and increasing with respect to $\theta$ $(10^{\circ }\le \theta \le 40^{\circ })$ and afterwards slightly decreasing again when $\theta \ge 40^{\circ }$ till grazing incidence. One can observe from Fig. 9b the Hall current’s effect on $|z_{2}|$ by taking $\alpha =0.003, e_{1}=0.02, \omega =655$. Further, it is supposed that the solid is rotating with $\Omega =0.007$. For different values of the Hall current parameter, the curve is first increasing with respect to $\theta$ $(0^{\circ } \le \theta \le 45^{\circ })$ and afterwards, it is decreasing for $45^{\circ } \le \theta \le 80^{\circ }$ beyond the $\theta =80^{\circ }$ smooth curve has been observed. We also observed that the curves are moving down with increasing values of the Hall current parameter. It is significant to note that the amplitude ratio of the *qT* wave is decreasing with increasing Hall current parameter’s values. It indicates that the amplitude ratio is dependent on the Hall current parameter. We can see the variation of $``m''$ on $|z_{3}|$ as shown in Fig. 9c. It can be seen that when the Hall current parameter’s values increases the amplitude ratio of the *qSV* wave decreases $(0^{\circ } \le \theta \le 10^{\circ })$ afterwards, increasing with increasing values of $``m''$ $(\theta \ge 10^{\circ })$ till the grazing angle of incidence. Amplitude ratio is increasing with respect to $\theta$ $(0^{\circ }\le \theta \le 10^{\circ })$ and decreasing with respect to $\theta$ when $(\theta \ge 10^{\circ })$ till the grazing angle of incidence. Amplitude ratio has its peak value at $\theta =10^{\circ }$. Finally, at $\theta =\frac{\pi }{2}$, the amplitude ratio decreases to zero. The variation of $``m''$ on amplitude ratio of the quasi void wave is shown in Fig. 9d. The amplitude ratio of the *qV* wave is decreasing when the Hall current parameter is increasing. We can see that the amplitude ratio decreasing with increasing values of $\theta$. Finally, at grazing incidence angles, the amplitude ratio decreases to zero.

**Figure 10:**
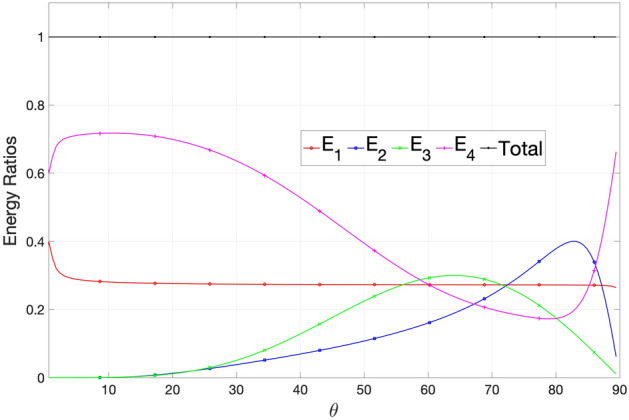
Energy ratio of waves versus angle of incidence.

In Fig. 10 one can observe that the *qP* wave consumes 40% energy at normal incidence then it consumes energy. It shows that the *qT* wave consumes maximum energies at $\theta =83^{\circ }$ and the *qSV* wave consumes energy at $\theta =65^{\circ }$, which are respectively 40% and 30% and then energy consumption decreases till grazing incidence. The sum of all the energy ratios, i.e, $SUM= E_{1}+E_{2}+E_{3}+E_{4}$ is shown by the solid black line at unity, indicating that the total energy is 100% conserved over the whole range of incident angles.

## Concluding remarks

This investigation relates to the research on Hall current on propagation and reflection of elastic waves through a non-local fractional-order thermoelastic rotating medium with voids. The system is split up into longitudinal and transverse components using the Helmholtz vector rule. It is observed that, through the frequency dispersion relation, four coupled quasi-waves exist in the medium. Dispersive waves are also observed. Analytically, the reflection coefficients of the wave are computed. The amplitude ratio and the speed of propagation of the wave are plotted graphically for rotational frequency, nonlocal, fractional order, and Hall current parameter. The cut-off frequency of the waves is also presented graphically. The energy conservation law is proved in the form of energy ratios. The earlier findings in the literature are obtained as special case in the absence of rotation, Hall current parameter, and porous voids. The graphs show that the following observations are listed as significant points:

- All the propagating waves are dispersive, as they depend on angular frequency. The quasi-longitudinal wave *qP* and quasi-transverse wave *qSV* show cut-off frequencies. The nonlocal parameter effects all the waves except the void wave.
- All the waves are dependent on the rotational frequency parameter. *qP* and *qSV*-waves show cut-off frequency, but *qT* and *qV*-waves show decreasing behavior. All four waves are dependent on the fractional order parameter. The *qP* wave shows cut-off frequencies, the *qT* wave is decreasing, and *qSV* wave has mix type behavior. The spikes in propagation speed of the *qV* wave shifted close to the origin for lower values of fractional order and shifted away from the origin for higher values.
- The Hall current parameter tends to decrease the speed of the *qT* and *qV* waves. However, *qP* and *qSV* waves show decreasing behavior, *qP* wave has the same effect beyond the cut-off frequency, but *qSV*-wave has opposite effect beyond the cut-off frequency.
- The amplitude ratios of *qP* and *qSV*-waves are decreasing with increasing values of nonlocal parameter. However, the amplitude ratios of the *qT* and *qV*-waves are increasing.
- The amplitude ratios of the *qT* and *qSV* waves are increasing with respect to rotational frequency. The amplitude ratio of the *qP* wave is affected randomly depending upon the angle of incidence by the rotation of the medium.
- The fractional order parameter strongly effects the amplitude ratios of the *qP* and *qT* waves. Both waves are dependent on the fractional order parameter. The *qSV* wave is decreasing and *qV* wave is increasing with respect to the fractional order parameter.
- The amplitude ratios of the *qT* and *qV* waves are decreasing with Hall current parameter. But the amplitude ratio of the *qP* and *qSV* waves are affected randomly.

The present investigations may find wide range of applications in various fields including engineering, the petroleum industry, material science, biology, and the study of the behavior of sound-absorbing materials, use porous materials. The current work is aimed to investigate the plane waves propagation through nonlocal in the context of thermoelastic rotating medium through porous medium.

## Data Availability

All data generated or analysed during this study are included in this published article.
